# Measuring Burden of Diseases in a Rapidly Developing Economy: State of Qatar: National Burden of Diseases in Qatar

**DOI:** 10.5539/gjhs.v5n2p134

**Published:** 2012-12-21

**Authors:** Abdulbari Bener, Mahmoud A. Zirie, Eun-Jung Kim, Rama Al Buz, Mouayyad Zaza, Mohammed Al-Nufal, Basma Basha, Edward W Hillhouse, Elio Riboli

**Affiliations:** 1 Department of Medical Statistics & Epidemiology. Hamad Medical Corporation, Department of Public Health Weill Cornell Medical College, Qatar; 2 Dept. of Evidence for Population Health Unit, School of Epidemiology and Health Sciences, The University of Manchester, Manchester, UK; 3 Endocrinology Unit, Dept. of Medicine, Hamad Medical Corporation, Weill Cornell Medical College, Qatar; 4 Department of Nursing, Cheju Halla University, Korea; 5 Acting Chief for Medical & Academic and Research Affairs, Chief Policy Advisor on Academic Health Systems, Hamad Medical Corporation, Qatar; 6 School of Public Health, Cancer Epidemiology & Prevention, Imperial College, London, UK

**Keywords:** DALY, YLD, YLL, disability, mortality, incidence

## Abstract

**Background:**

The Global Burden of Disease (GBD) study has provided a conceptual and methodological framework to quantify and compare the health of populations.

**Aim:**

The objective of the study was to assess the national burden of disease in the population of Qatar using the disability-adjusted life year (DALYs) as a measure of disability.

**Methods:**

We adapted the methodology described by the World Health Organization for conducting burden of disease to calculate years of life lost due to premature mortality (YLL), years lived with disability (YLD) and disability adjusted life years (DALYs). The study was conducted during the period from November 2011 to October 2012.

**Results::**

The study findings revealed that ischemic heart disease (11.8%) and road traffic accidents (10.3%) were the two leading causes of burden of diseases in Qatar in 2010. The burden of diseases among men (222.04) was found three times more than of women's (71.85). Of the total DALYs, 72.7% was due to non fatal health outcomes and 27.3% was due to premature death. For men, chronic diseases like ischemic heart disease (15.7%) and road traffic accidents (13.7%) accounted great burden and an important source of lost years of healthy life. For women, birth asphyxia and birth trauma (12.6%) and abortion (4.6%) were the two leading causes of disease burden.

**Conclusion::**

The results of the study have shown that the national health priority areas should cover cardiovascular diseases, road traffic accidents and mental health. The burden of diseases among men was three times of women's.

## 1. Introduction

The burden of disease in a country refers to the assessment of mortality, morbidity, injuries, disabilities and other risk factors specific to that country. Monitoring the health status of the population is one of the core functions of the government, health authorities and other public health agencies. In order to evaluate the burden of disease and health care performance, a scientific measure is needed that can be used to quantify health status and to create a systematically organized classification system capable of including every important disease entity (Lopez & Murray, 1998). Mortality data are the most widely used source of information for identifying most important health problems for a population. Mortality data is an inadequate measure of the health of a population because of two reasons that death rates in economically developed countries have fallen substantially during the 20^th^ century and many people can live for years with serious illness and disability (Lopez et al., 2006). Hence, the World Health Organization (WHO), World Bank and the Harvard School of Public Health researchers developed the disability-adjusted life-years (DALYs) (Murray & Lopez, 1996), a new metric for measuring the burden of disease. DALYs combine number of deaths, impact of premature death, and disability to establish a burden of disease measure of health status. DALYs is a single measurement that consists of a summation of years of healthy life lost as a result of premature death and time lived under disabling conditions (McKenna, Michaud, Murray, & Marks, 2005). This burden of disease indicator can be used to judge progress over time within a single country or region. Many countries have adopted this approach as the standard for health accounting and for guiding the determination of health research priorities.

The remarkable changes in demographic and epidemiological factors and risk patterns across the world over the last decades have had a significant impact on the population health status. The epidemiological transition from infectious to chronic non-communicable disease in this group of countries is of major relevance to health planning. Developing countries are facing a triple burden of disease from communicable diseases, non-communicable diseases and injuries. The State of Qatar has undergone major social and economic changes resulting in rapid urbanization and huge increase in the use of private transport vehicles with a consequent increase in the burden of chronic disease and injuries (Bener et al., 2008). Like other high income countries, the burden of disease in the Middle Eastern region including Qatar is becoming increasingly dominated by chronic, non-communicable conditions and injuries. Since 1993, many countries have conducted burden of disease studies at international and national level using DALYs (Ljung, Peterson, Hallqvist, Heimerion, & Diderichsen, 2005; Mathers, Vos, Stevenson, & Begg, 2001; Lopez & Murray, 1998; Hyder, Rotllant, & Morrow, 1998). Because of the growing importance of non-communicable diseases and their often non-fatal impact on health, it was important to conduct the burden of Disease (NBoDQ) study in Qatar to understand the magnitude of the major health problems. The national burden of study (NBoDQ), which was the first study of its type, has assessed the leading causes of burden of disease in Qatar using disability-adjusted life years (DALYs) as measures of disability.

## 2. Methodology

The burden of disease study in Qatar (NBoDQ) was conducted during the period November 2011 to October 2012. The study applied the methods developed for the original Global Burden of Disease study to data specific to Qatar to compute the DALYs (Murray and Lopez, 1996). To assess burden of disease, GBD used a time-based metric that measures both premature mortality (Years of life lost because of premature mortality or YLL) and disability (Years of healthy life lost as a result of disability or YLD, weighted by the severity of the disability). The disease classification is based on the Global Burden of Disease as defined by WHO and adheres to the principle that a disease classification should be exhaustive and exclusive. We collected the mortality and morbidity data and addressed the disease classifications used by WHO and other DALYs yielding procedures. The characteristics of the data sources used to estimate DALYs were scrutinized. The data concerning the disease structure in Qatar such as major causes of death and frequent medical service treatment diseases were investigated. Mortality data for the year 2010 according to ICD 10, by age group were collected from the vital statistics database. Also, life expectancy table for the year 2007 was used. For the disability data and for the majority of conditions, the number of incident cases was available directly from disease registries, routine databases and epidemiological research surveys. We have used the inpatient discharge abstract of the hospitals, Cancer disease registry and some of the community surveys for the incidence of other diseases. Where sufficient data were not available to run the model, the incidence estimates developed in other studies were used like mental disorders and injuries. The complete details of each minor and major category of ICD have been mentioned with its frequency, length of stay and number of deaths. Similarly, we have done the analysis for the year 2000 to compare the results and assess the trend on leading burden of diseases in Qatar.

Using DALYs, World Health Organization arranged ICD codes into 3 major groups. In group 1, communicable diseases like herpes genitals, cholera, typhoid and paratyphoid, shigellosis, mumps, rubella, chicken pox, pneumonia, influenza …etc were included. In group 2, non-communicable diseases such as neoplasms, endocrine, nutritional, metabolic disorders, circulatory, respiratory, digestive system diseases, injuries were included. Neuro-psychiatric diseases were included in group 3.

DALY Estimation: DALY is a summary measure that represents health status. The sum of the two components, DALYs, provides a measure of the future stream of healthy life (years expected to be lived in full health) lost as a result of the occurrence of specific disease and injuries. The effect of fatal case (of disease or injuries) is captured by years of life lost, while YLD captures the future health consequences in terms of diseases or injuries of incident cases that were not fatal. DALYs were obtained from the addition of two components:

DALY = Years of life lost (YLL) + Years of life lived with disability (YLD)

Years of life lost (YLL) were calculated by multiplying age-specific mortality rates by age-specific standard expected YLL and population numbers. For most conditions YLD were estimated by multiplying age-specific incidence rates by average duration of each incident case (or more precisely, of the associated disability until death or recovery) and average disability weight.

Ethical approval was obtained from the Hamad Medical Corporation Institutional Review Board for conducting this research in Qatar.

## 3. Results

As shown in Table 1, the leading causes of disease burden among the population (DALYs/100,000 persons) was Ischemic heart disease (11.8%), followed by road traffic accidents (10.3%), birth asphyxia and birth trauma (7.8%), cerebrovascular diseases (4.5%) and mental disorders (2.9%). Road traffic accidents resulted in the greatest number of years of life lost (YLL) (30.84), while ischemic heart disease caused the greatest life disability (YLD) at 32.72. The leading causes of disability in Qatar included three types of injuries such as road traffic accidents (10.3%), self inflicted injuries (2.3%) and falls (1.6%), for which direct mortality was high.

**Table 1:** Disability adjusted life years (DALYs), total years of life with disability (YLD), Years of life lost and percent of total DALYs, 2010

DALY Rank	Disease	DALY per 100,000 persons	YLD per 100,000 persons	YLL per 100,000 persons	Percent of Total DALYs.
1	Ischemic heart disease	35.26	32.72	2.54	11.8%
2	Road traffic accidents	30.84	0.00	30.84	10.3%
3	Birth asphyxia and birth trauma	23.36	23.36	0.00	7.8%
4	Cerebrovascular disease	13.41	10.08	3.33	4.5%
5	Mental disorder	8.50	5.94	2.56	2.9%
6	Self-inflicted injuries	6.96	0.00	6.96	2.3%
7	Appendicitis	5.97	5.97	0.00	2.0%
8	Falls	4.65	0.00	4.65	1.6%
9	Endocrine disorders	4.08	2.52	1.56	1.4%
10	Abortion	3.32	3.32	0.00	1.1%
11	Diabetes mellitus	2.70	1.86	0.84	0.9%
12	Lower respiratory infections	2.08	0.81	1.26	0.7%
13	Hypertensive heart disease	1.68	1.30	0.39	0.6%

As shown in Table 2, unipolar depressive disorders (6.7%) ranked as the first leading cause of disease in the population in the year 2000. Road traffic accidents (6.6%) were the second followed by ischaemic heart disease (5.4%). Ischemic heart disease (11.6%) together with road traffic accidents (11.3%) and lower respiratory infections (10.2%) were the leading causes of premature mortality (YLL), whereas unipolar depressive disorders (12%), hearing loss (7.1%) and diabetic mellitus (4.1%) were found to be the leading causes of disability.

**Table 2:** Leading causes of burden of diseases (% of total DALYs), mortality (% of total YLL) and disability (% of total YLD) in 2000

DALY Rank	Disease	Percent of total DALYs	Percent of total YLL	Percent of total YLD
1	Unipolar depressive disorders	6.7	0.0	12.0
2	Road traffic accidents	6.6	11.3	2.9
3	Ischemic heart disease	5.4	11.6	-
4	Lower respiratory infections	5.2	10.2	-
5	Hearing loss, adult onset	4.0	-	7.1
6	Diabetes mellitus	3.8	3.3	4.1
7	Conditions arising during the perinatal period	3.4	5.1	-
8	Congenital anomalies	2.8	3.6	-
9	Drug use disorders	2.2	-	3.9
10	Hypertensive heart disease	2.0	4.4	-
11	Self-inflicted injuries	-	2.6	-
12	Cerebrovascular disease	-	2.3	-
13	Breast cancer	-	1.8	-

As shown in Table 3, the leading four causes of DALYs for men were Ischemic heart disease (15.7%), road traffic accidents (13.7%), birth asphyxia and birth trauma (6.5%), and cerebrovascular disease (5.8%).

**Table 3:** Disability adjusted life years (DALYs) and percent of total DALYs for men, all ages, 2010

DALY rank	Disease	DALY per 100,000 persons	Percent of Total DALY
1	Ischemic heart disease	34.97	15.7%
2	Road traffic accidents	30.44	13.7%
3	Birth asphyxia and birth trauma	14.34	6.5%
4	Cerebrovascular disease	12.81	5.8%
5	Mental disorder	11.8	4.8%
6	Self-inflicted injuries	6.91	3.1%
7	Appendicitis	5.78	2.6%
8	Falls	4.55	2.1%
9	Endocrine disorders	3.17	1.4%
10	Diabetes mellitus	2.55	1.1%
11	Lower respiratory infections	1.95	0.9%
12	Hypertensive heart disease	1.59	0.7%
13	Trachea, bronchus, and lung cancers	1.21	0.5%

As shown in Table 4, the first cause was birth asphyxia and birth trauma (12.6%), followed by abortion (4.6%) and hypertensive disorders of pregnancy (2.3%) in women. Mental disorders including phobia and unipolar depressive disorders are also important causes of lost years of healthy life for women in Qatar.

**Table 4:** Disability adjusted life years (DALYs) and percent of total DALYs for women all ages, 2010

DALY rank	Disease	DALY per 100,000 persons	Percent of Total DALY
1	Birth asphyxia and birth trauma	9.02	12.6%
2	Abortion	3.32	4.6%
3	Hypertensive disorders of pregnancy	1.62	2.3%
4	Maternal hemorrhage	0.99	1.4%
5	Mental disorder	0.97	1.2%
6	Endocrine disorders	0.91	1.3%
7	Cerebrovascular disease	0.60	0.8%
8	Breast cancer	0.54	0.8%
9	Obstructed labor	0.47	0.7%
10	Epilepsy	0.41	0.6%
11	Unipolar depressive disorders	0.40	0.6%
12	Road traffic accidents	0.40	0.6%
13	Colon and rectal cancers	0.30	0.4%
14	Phobia	0.29	0.4%
15	Ischemic heart disease	0.29	0.4%

**Figure 1:**
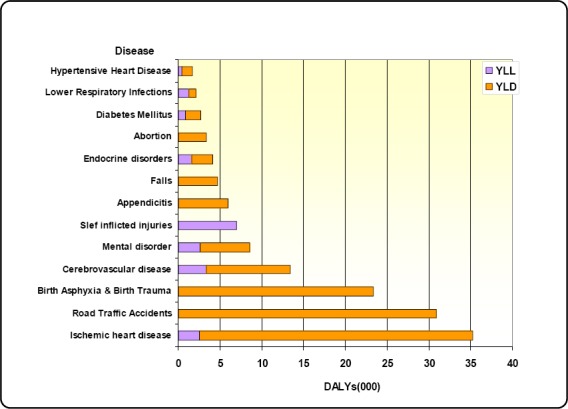
Burden of diseases (Years lived with disability (YLD) and Years of life lost (YLL) in 2010

The burden of disease (years lived with disability (YLD) and years of life lost (YLL) in 2010. For ischemic heart disease, the contribution to YLD was higher than that to YLL. The burden of road traffic accidents, birth asphyxia and birth trauma, appendicitis and falls were mainly due to lengthy periods of disability, whereas self inflicted injury was totally due to premature death (YLL).

**Figure 2:**
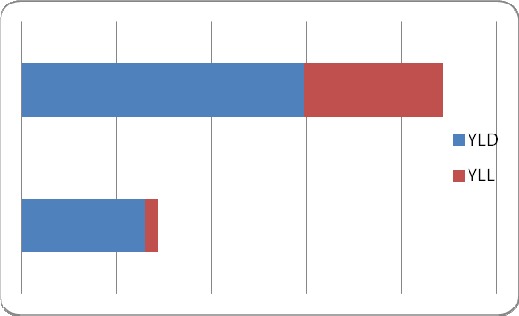
Gender distribution of burden of disease, 2010 (unit DALY/100,000populations)

As shown in Figure 2, the gender distribution of burden of disease in 2010. Men bear a much greater burden of disease (222.04) than women (71.85) and this is associated with an increased premature death rate.

**Figure 3:**
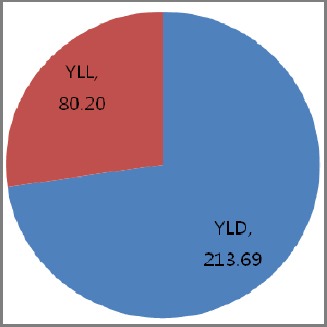
Total burden of disease in 2010 of Qatar

As shown in Figure 3, total burden of disease across all diseases in 2010 in Qatar. The total DALYs per 100,000 populations was 293.89 per 100,000 populations. Of which, 72.7% was due to non fatal health outcomes and 27.3% was due to premature death.

**Figure 4:**
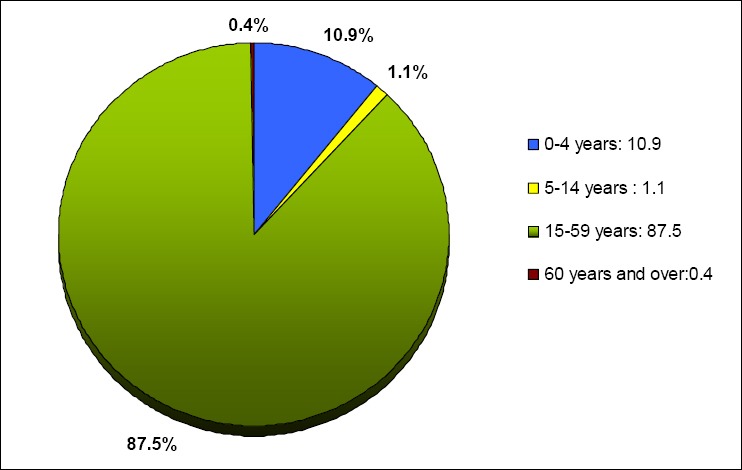
Age distribution of burden of disease, 2010

Out of total DALYs, 12% of the total disease and injury burden occurred in children below 15 years and 87.5% in adults aged 15-50 years.

**Figure 5:**
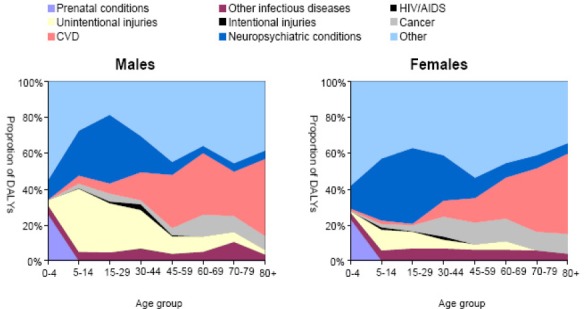
Proportion of DALYs by Cause, age group and sex for the year 2000

## 4. Discussion

This is the first study to identify the most important health problems in Qatar using DALY, which combines information on both the impact of premature death and the impact of disability. Global burden study (Murray, Lopez, & Jamison, 1994) confirmed that disability and states of impaired health caused by diseases and injuries play a central role in determining the overall health status of populations in all regions of the world. Since 1993, many countries have conducted burden of disease studies at international and national level using DALY, which compared disease burden across a range of diseases, injuries and risk factors. In Qatar, the present national burden of study (NBoDQ) revealed that non communicable diseases and road traffic accidents were a significant cause of health burden in Qatar (Bener, Ozkan, & Lajunen, 2008), as it was revealed in all regions (Murray & Lopez, 1996; Murray et al., 1994; Begg et al., 2007; Lopez, Mathers, Ezzati, Jamison, & Murray, 2006). The burden of non-communicable diseases is now growing rapidly in Qatar as a consequence of population ageing, major changes in lifestyle and socio-economical characteristics that caused a major shif in the distribution of diseases risk factors.

The results obtained from the DALY calculation reveal that the burden of disease in Qatar originates primarily from ischemic heart disease, road traffic accidents and birth asphyxia and birth trauma, as these diseases were the major causes of lost years of healthy life as measured by DALYs. Ischaemic heart disease was also the major cause of total DALYs for both genders in Australia (Mathers, Theo, Stevenson, & Begg, 2000), U.S (Mckenna et al., 2005) and Serbia (Jankovic et al., 2006). Cardiovascular disease (20%), cancers (19%) and mental disorders (14%) were the three leading causes of overall burden in Australia (Mathers et al., 2000). Our findings in this regard are comparable with these countries, but different from similar studies conducted in Korea (Yoon et al., 2007) and Iran (Naghavi et al., 2009). In Iran, injuries (28%) were found to be the most prominent cause of disease burden, followed by mental disorders (16%) and circulatory diseases (10%). In Korea (Yoon et al., 2007), however, the three leading causes of increased DALY rates were cancer, cardiovascular diseases and digestive diseases. It is important to note that seven of the leading causes of burden of disease within the 13 DALY ranks in Qatar appeared among the top twenty causes of burden of disease globally (Hyder, Rotland, & Morrow, 1998). This shows that the leading causes of burden of disease in Qatar were broadly similar to those for the world in 2004 apart from diarrhoeal diseases, HIV/AIDS, prematurity and low birth weight and neonatal infections.

Using DALY as a measure, the total disease burden was highest for ischemic heart disease and road traffic injuries in many developed and developing countries (Mathers et al., 2000). Also, the 2004 Global burden of disease report (Hyder et al., 1998) showed that ischemic heart disease is the fourth leading cause of disease burden globally with road traffic accidents in 9^th^ place. In Qatar, between the years 2000 and 2010 the burden of disease from ischemic heart disease has increased from the 3^rd^ to 1^st^ position, with road traffic accidents maintaining 2^nd^ position. Interestingly in the current study, ischemic heart disease accounted for 11.8% of total DALYs compared to (5.4%) in 2000. Also, the proportion of road traffic accidents increased from 6.6% in 2000 to 10.3% in 2010. Diabetes mellitus was one of the leading cause of disease burden in both 2000 and 2010. This is consistent with our previous reports (Bener et al., 2009); (Bener, Zirie, Musallam, Khader, & Al-Hamaq, 2009), which showed a high prevalence of diabetes mellitus (DM) in the adult Qatari population. Interestingly these studies found high proportion of pre-diabetes in adult population, which would increase the prevalence of DM in the future. This finding is quite alarming and should inform the priority setting in public health policies and research.

The state of Qatar has experienced a rapid transition in its socioeconomic status and people in Qatar enjoy a high standard of living, which has resulted in a substantial improvement in their life expectancy and their lifestyle. The high prevalence of ischemic heart disease is probably linked to the high prevalence of obesity and diabetes mellitus type 2, limited physical activity and the excessive consumption of saturated fats. Also, it has been found that the economic growth and the changes in the mode of transportation is associated with the increase in the number of traffic deaths as reported in a study of Bener et al. (2008). The previous studies (Bener et al., 2008; Bener, Al suwaidi, al-Jaber, Al-Marri, & Elbagi, 2004) supported the higher DALY rates of ischemic heart diseases and road traffic accidents. Similar results were found in the Global Burden of Disease report (Hyder et al., 1998) where ischaemic heart disease was the 2^nd^ DALY rank in the middle and high income countries, whereas the road traffic accidents was the 2^nd^ leading disease in middle income countries and 10^th^ rank in high-income countries.

In Qatar, the total burden of disease in 2010 was 293.9 per 100,000 population. 72.7% of the total DALY was due to non-fatal health outcomes (YLD) and 27.3% was due to premature deaths (YLL). This finding of YLD and YLL contributions to total DALYs is consistent with other similar assessments in a developed country like Serbia (78%: 22%). Years of life lost to premature mortality (YLL) and years of life lost due to disability (YLD) for burden of disease groups revealed that road traffic injuries exhibited the highest years of life lost (YLL) (30.84), while ischemic heart disease was the highest contributor to years of life disability (32.72) in Qatar. The opposite was found in Australia where Ischemic heart disease (20.5%) was the largest cause of years of life lost (YLL) and mental disorders was the leading cause of YLD (30%). An entirely different pattern was observed in Korea where cancer was determined to be the leading cause of premature mortality (YLL) and digestive diseases were found to be the leading cause of disability (YLD).

With regard to the gender distribution of burden of disease in 2010, the majority burden of disease in men was three times that of women. The mortality burden (YLL) and disability burden (YLD) were larger for men (YLL) than for women in Qatar. Interestingly 75.5% of the total population is men and most of them are working force, mainly bachelors in the age group 20 – 50 years. This may well explain the big gap in burden of disease between men and women. In contrast almost an equal burden of disease for gender was observed in Iran (Naghavi et al., 2009) with 53% for males and 47% for females. In Qatar, the DALY rank of total burden of disease across all diseases appeared to be the same DALY rank order among men.

In Qatar, the male burden is higher for ischemic heart diseases (15.7%), road traffic accidents (13.7%) and birth asphyxia and birth trauma (6.5%). The leading causes of DALYs for men in Serbia were ischemic heart disease, road traffic accidents and self-inflicted injuries, which is comparable without study results. The non communicable diseases including mental disorders made up 5 out of 14 leading causes of disease burden in men. A high prevalence of mental disorders was reported in previous studies of (Bener, Al-Kazaz, Ftouni, Al-Harathy, & Dafeeah, 2012; Bener, Ghuloum, & Abou-Saleh, 2012). Injuries accounted for 18.9% of the diseases burden in men. Mental disorders (4.8%) and self-inflicted injuries (3.1%) were higher in men compared to women. Self-inflicted injuries account for an increasing share of the burden and are highly relevant to mental disorder life depressions. In men, mental disorders or unipolar depressive disorders are major cause of disease burden. Even in Australia (Mathers et al., 2000), it was reported that males have a greater incidence of the major diseases and injuries associated with high case fatality. In Korea (Yoon et al., 2007), Diabetes mellitus and cardiovascular disease were exhibited the highest DALY score.

In Qatar, maternal conditions are a major contributor to the high burden of disease for women. The burden of reproductive problem (21.6%) is so great that maternal conditions make up 5 out of 15 leading causes of disease burden in women. These results are in agreement with other studies conducted in Qatar showing that maternal complications were high in pregnant women with a consequent increased of neonatal morbidity and mortality (Bener, Salameh, Yousafzai, & Saleh, 2012; Nimeri et al., 2012; Bener et al., 2012; Bener, Saleh, & Al-Hamaq, 2011; Rahman et al., 2011). Mental disorders including phobia and unipolar disorders are also an important source of lost years of healthy life for women in Qatar. In developed countries like Australia (Mathers et al., 2000) and U.S. (Mckenna et al., 2005), the burden of disease pattern was different in women since ischemic heart disease was the leading cause of disease burden. This increased burden of disease by women of reproductive age has not been reported as a major cause of healthy lost years in other countries. A greater emphasis on improved care for women during pregnancy and childbirth could make a large contribution to reducing the burden of disease.

The findings of this study indicate that most DALYs are attributed to non-communicable diseases in the Qatari population. It confirms the growing importance of non-communicable diseases and highlights important changes in population health. DALYs measure is an important tool to assess the population health and will assist in health planning and priority setting in the health sector.

## 5. Limitations

In certain diseases, data to assess burden of disease are not readily available. No ongoing comprehensive electronic health registry exists to assess the morbidity burden at the national level. Mortality, communicable diseases, and inpatient discharge diagnosis have been classified according to ICD 10 and the data are available electronically. We have used these databases and incidences in the community surveys to run the model for measuring the burden of disease. But, visits to outpatient clinics of hospitals are not classified according to ICD which leads to underestimate of incidence of diseases.

## 6. Conclusion

The study findings revealed that the major causes of lost years of healthy life was primarily from ischemic heart disease, road traffic accidents and birth asphyxia and birth trauma. With regard to sex differentials for leading causes of DALYs, the burden of majority of diseases was higher for men. Ischemic heart disease, road traffic accidents and birth asphyxia and birth trauma made an important contribution to the burden of disease in men, while reproductive problems were the leading causes of disease burden in women. The DALY results have shown that the national health priority areas should cover cardiovascular diseases, road traffic accidents and mental health.
